# Combined Therapy of Iron Chelator and Antioxidant Completely Restores Brain Dysfunction Induced by Iron Toxicity

**DOI:** 10.1371/journal.pone.0085115

**Published:** 2014-01-06

**Authors:** Jirapas Sripetchwandee, Noppamas Pipatpiboon, Nipon Chattipakorn, Siriporn Chattipakorn

**Affiliations:** 1 Neurophysiology Unit, Cardiac Electrophysiology Research and Training Center, Department of Physiology, Faculty of Medicine, Chiang Mai University, Chiang Mai, Thailand; 2 Department of Oral Biology and Diagnostic Science, Faculty of Dentistry, Chiang Mai University, Chiang Mai, Thailand; Alexander Fleming Biomedical Sciences Research Center, Greece

## Abstract

**Background:**

Excessive iron accumulation leads to iron toxicity in the brain; however the underlying mechanism is unclear. We investigated the effects of iron overload induced by high iron-diet consumption on brain mitochondrial function, brain synaptic plasticity and learning and memory. Iron chelator (deferiprone) and antioxidant (n-acetyl cysteine) effects on iron-overload brains were also studied.

**Methodology:**

Male Wistar rats were fed either normal diet or high iron-diet consumption for 12 weeks, after which rats in each diet group were treated with vehicle or deferiprone (50 mg/kg) or n-acetyl cysteine (100 mg/kg) or both for another 4 weeks. High iron-diet consumption caused brain iron accumulation, brain mitochondrial dysfunction, impaired brain synaptic plasticity and cognition, blood-brain-barrier breakdown, and brain apoptosis. Although both iron chelator and antioxidant attenuated these deleterious effects, combined therapy provided more robust results.

**Conclusion:**

In conclusion, this is the first study demonstrating that combined iron chelator and anti-oxidant therapy completely restored brain function impaired by iron overload.

## Introduction

Iron acts as an essential element for cellular processes in several organs, including the central nervous system [1]. In the brain, iron is required for brain function such as myelination [2], neurotransmitter synthesis [3], nitric oxide metabolism [4], and other biochemical activities [5]. Although the brain relies on iron availability for many important functions, it is also a highly vulnerable organ to iron-induced oxidative stress. Excessive iron accumulation or iron overload in the brain can be found in a normal aging brain [6], or under the pathologic alterations of iron homeostasis [7]. Iron overload is commonly found in beta-thalassemic patients with regular blood transfusions [8], [9]. The major organ dysfunction related to hemosiderosis is principally hepatic cirrhosis and cardiomyopathy [10], [11]. However, little is known regarding iron overload due to chronic blood transfusion associated with brain dysfunction. It has been known that the regulation of iron in the brain, particularly in the hippocampus is crucial for learning and memory [12], [13]. A previous study in beta-thalassemic patients with iron overload demonstrated the potential role of hemosiderosis on cognitive impairment [14], [15]. Although synaptic plasticity is an important process in learning and memory [16], its association with iron overload in the brain is unclear. A recent study also suggested the beneficial role of iron in N-methyl-D-aspartate receptor-dependent stimulation of calcium-induced brain synaptic plasticity [17]. This finding suggested that iron was required for synaptic plasticity. However, the synaptic plasticity under an iron overload condition has rarely been studied. In addition, the underlying mechanism of iron overload onto the brain synaptic plasticity as well as learning and memory has not yet been investigated.

Mitochondria are vital organelles, which produce energy for cells, particularly in the brain [18]. In fact, iron could act as a cofactor in mitochondria for the oxidative phosphorylation process [5]. Excessive iron accumulation could affect brain mitochondrial function resulting in neurodegeneration [19]. Therefore, it is possible that systemic iron overload may cause brain iron accumulation, leading to impaired brain mitochondrial function, brain apoptosis, and eventually the disruption of learning and memory. However, this possibility has not yet been investigated.

Oxidative stress has been proposed as one of the major contributing factors in iron-overload brain [20]. To protect against the deleterious effects of brain iron accumulation, potential strategies are to protect the brain from iron-induced oxidative stress. N-acetyl cysteine (NAC) has been commonly known as an antioxidant agent. Several studies have shown that NAC attenuated the oxidative stress by reducing reactive oxygen species (ROS) production in the retinal blood vessels and the heart of rat models [21], [22].

Besides anti-oxidative strategy, iron chelator has been commonly used to prevent iron-overload induced organ dysfunction [23], [24]. The iron chelators, including deferiprone (L1), or deferoxamine (DFO), are widely used to chelate iron overload in thalassemic patients. Several studies have shown that iron chelation prevents the deleterious effects of iron accumulation in the brain during brain injury [25], [26], suggesting that iron chelation can be a potential strategy for protection from neurodegenerative disorders during iron overload. However, the effects of either deferiprone, NAC or the combined agents on the brain under iron-overload conditions have not yet been investigated.

In the present study, we investigated whether iron overload induced by long-term high iron diet (HFe) consumption could lead to brain iron accumulation, decreased brain synaptic plasticity, impaired learning and memory as well as brain mitochondrial dysfunction and brain apoptosis. In addition, we determined the effects of antioxidant agent as well as an iron chelator on synaptic plasticity, learning and memory and brain mitochondrial function in HFe-fed rats. We hypothesized the following: 1) Iron overload condition induced by long-term HFe consumption causes plasma oxidative stress, leading to blood brain barrier breakdown, allowing iron to enter and accumulate in the brain, thus leading to brain oxidative stress, 2) Iron overload causes brain mitochondrial dysfunction, brain apoptosis, impaired synaptic plasticity, and learning and memory deficit, 3) Pharmacological interventions with deferiprone and NAC attenuate the iron overload induced brain dysfunctions and 4) Combined therapy of deferiprone and NAC restores brain functions after iron overload induced by long-term HFe consumption.

## Materials and Methods

### Animal Preparation

All animal studies were approved by the Institutional Animal Care and Use Committee (IACUC) at the Faculty of Medicine, Chiang Mai University (Permit number: 06/2556). Adult male Wistar rats (120–160 g, n = 66) were obtained from the National Laboratory Animal Center, Mahidol University, Bangkok, Thailand. All experiments were conducted in accordance to ARRIVE guidelines. They were housed in a room maintained under constant environmental conditions (temperature 22–25°C and a constant 12-hour light/dark cycle).

### Experimental Protocols

The experimental protocols were set to determine the effects of iron overload induced by long-term HFe consumption on peripheral parameters and brain functions, and the effects of pharmacological intervention on iron-overload induced brain dysfunctions.

**Figure 1:**
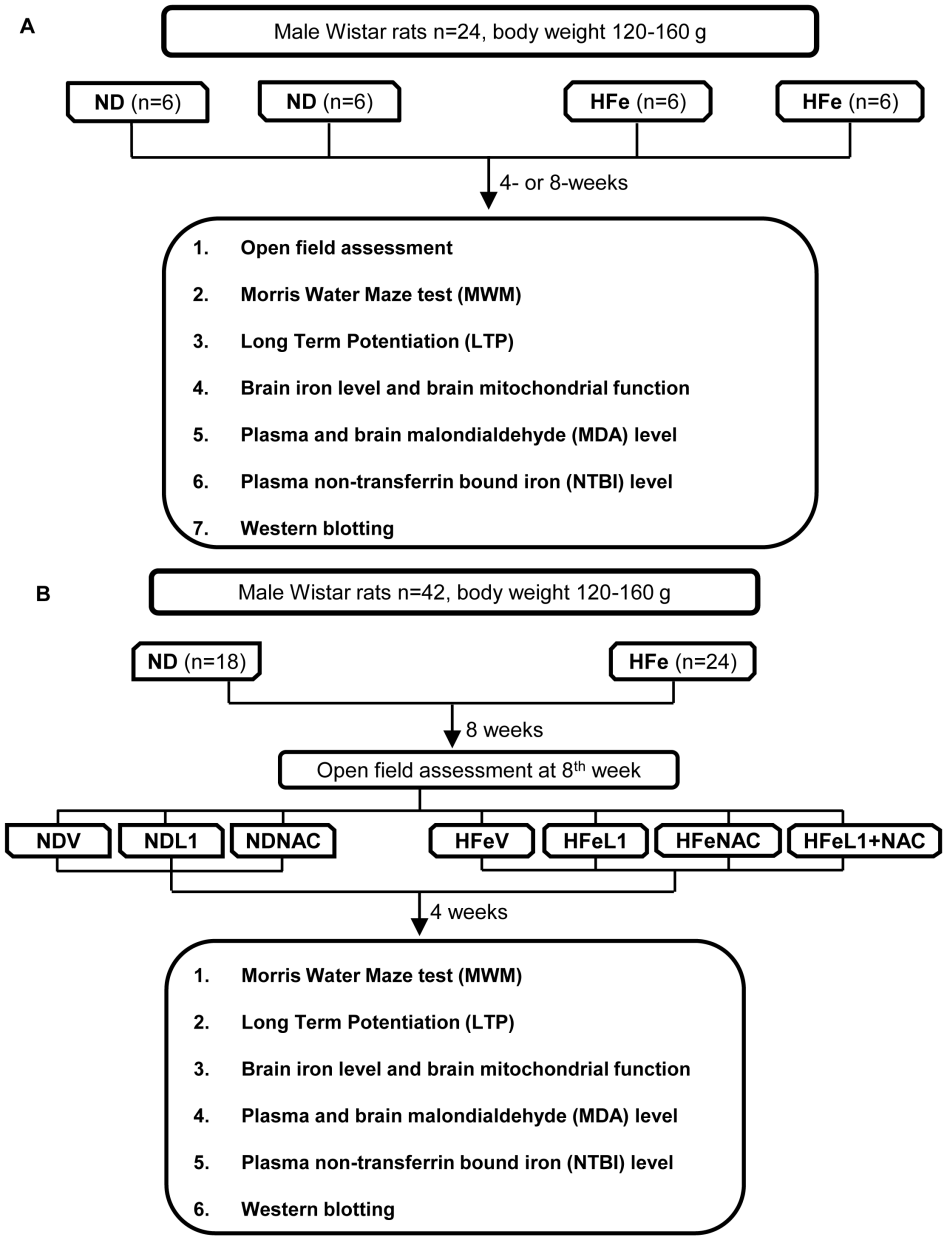
Summarized experimental diagrams. Each panel represented protocol 1 (A) and protocol 2 (B). ND: normal diet fed group; HFe: high iron diet fed group; MDA: malondialdehyde; NTBI: non-transferrin bound iron; V: vehicle; L1: deferiprone; NAC: n-acetyl cysteine.

In the first protocol (Figure 1A), male Wistar rats were randomly divided equally to receive either a normal diet (0.018% Fe/kg of diet, ND) or High iron diet (0.2% Fe/kg of diet, HFe) (n = 12/group). Then, rats in each dietary group were divided into 2 subgroups to receive the assigned diet for either 4 weeks or 8 weeks (n = 6/subgroup). In the second protocol (see figure 1B), male Wistar rats were randomly assigned to receive either ND (n = 18) or HFe diet (n = 24) for 8 weeks. Then, rats in the HFe group were further divided into 4 subgroups (n = 6/subgroup), including 1) HFe treated with saline (HFeV), 2) HFe treated with NAC (HFeNAC), 3) HFe treated with L1 (HFeL1) and 4) HFe treated with L1 combined with NAC (HFeL1+NAC). Rats in the ND group were divided into 3 subgroups, including 1) ND treated with saline (NDV), 2) ND treated with NAC (NDNAC), and 3) ND treated with L1 (NDL1). NDV and HFeV subgroups received 0.9% normal saline solution (NSS) as a vehicle at 2 ml/kg/day. NDNAC and HFeNAC subgroups received NAC 100 mg/kg/day (Sigma-Aldrich, St Louis, MO, USA; dissolved in normal saline 2 ml/kg/day). NDL1 and HFeL1 subgroups received deferiprone (L1) 50 mg/kg/day (Government Pharmaceutical Organization, Bangkok, Thailand; dissolved in normal saline 2 ml/kg/day). HFeL1+NAC subgroup received L1 50 mg/kg/day combined with NAC 100 mg/kg/day (dissolved in normal saline 2 ml/kg/day). Each subgroup received the assigned treatment for 4 consecutive weeks by gavage feeding.

At the end of the experiment, rats were deeply anesthetized (3% isoflurane and intraperitoneal injection of 80 mg/kg thiopental) and decapitated, and the brain excepted cerebellum region was rapidly removed, and separated into 2 halves. The first half was used for brain slices preparation to determine the long-term potentiation (LTP) and western blot analysis. The second half was used for isolated brain mitochondria preparation or stored at −80°C for brain iron and malondialdehyde (MDA) determination. Furthermore, blood samples from tail veins were collected every month of each experiment, and kept in Na-heparin tube for determination of plasma non-transferrin bound iron (NTBI) and MDA levels. The summary of the experimental protocols are shown in figure 1.

### Quantification of Plasma Non-transferrin Bound Iron (NTBI)

To determine iron overload condition, plasma NTBI concentration was measured using nitrilotriacetic acid disodium salt (NTA) chelation/HPLC method [27]. Plasma was incubated with 80-mM NTA solution, pH 7.0 for 30 minutes at room temperature to produce Fe^3+^- (NTA)_2_ complex and then subsequently separated from plasma proteins via plasma mixture spinning with a membrane filter (Nanostep®, 30-kDa cut off, polysulfone type; Pall life Sciences, Ann Arbor, MI USA). Concentration of Fe^3+^- (NTA)_2_ complex representing NTBI in the ultrafiltrate was determined by a non-metallic HPLC system. NTBI concentration was calculated from a calibration curve made with differential iron concentrations of Fe^3+^- (NTA)_2_ in 80 mM NTA, pH 7.0 ranging 0–16 µM.

### Quantification of Plasma and Brain Malondialdehyde (MDA)

To determine oxidative stress condition, plasma and brain malondialdehyde (MDA) concentrations were measured by the HPLC method [28]. Brain tissues were homogenized in a phosphate buffer pH 2.8. Homogenated brain or plasma was mixed with 10% trichloroacetic acid (TCA) containing butylated hydroxytoluene (BHT) (50 ppm), heated at 90°C for 30 minutes, and then cooled down and consequently centrifuged at 6,000 rpm for 10 minutes. Supernatant was mixed with 0.44 M H_3_PO_4_ and 0.6% thiobarbituric acid (TBA) solution and then incubated at 90°C for 30 minutes. MDA levels were determined via the absorbance detected at 532 nm by the HPLC system, and were calculated directly from the standard curve, and reported as the MDA equivalent concentration (µM).

### Brain Iron Level Assay

Brain iron determination was performed using a method modified from a previous study [29]. A homogenated brain was precipitated in protein precipitated solution (1∶1). The mixed solution was heated at 95°C for 1 hour and cooled down to room temperature. The mixed sample was centrifuged at 8,200 *g* for 10 minutes. A supernatant was mixed in chromagen solution (1∶1). For blank, a supernatant was mixed with 1.5 mol/l sodium acetate containing 0.1% TGA. The sample was incubated for 30 minutes at room temperature. Absorbance of samples was determined using a microplate reader at 562 nm.

### Brain Slices Preparation

Brain slices (400 µm) were prepared using a vibratome (Vibratome Co., Saint Louis, MO) [30]. Briefly, after a 30-minutes post slice incubation in high-sucrose aCSF, the brain slices were transferred to a standard aCSF solution and saturated with 95% O_2_/5% CO_2_ (pH 7.4) for an additional 30 minutes at room temperature (22–24°C). Brain slices (n = 3 brain slices per animal) were used for extracellular recording.

### Extracellular Recording of Brain Slices for Long-term Potentiation (LTP)

Brain slices were transferred to a submersion recording chamber and continuously perfused at 3–4 ml/min with standard artificial cerebrospinal fluid (aCSF) warmed to 28–29°C. Field excitatory postsynaptic potentials (fEPSPs) was evoked by stimulating the Schaffer collateral-commissural pathway with a bipolar tungsten electrode, while the fEPSPs recordings were taken from the stratum radiatum of the hippocampal CA1 region with micropipettes filled with 2 M NaCl. Brain slices were perfused with aCSF and recorded as a baseline condition for 10 minutes. LTP was induced by delivering high-frequency tetani (high-frequency stimulation (HFS); 4 trains at 100 Hz; 0.5 s duration; 20 s interval) at 1.5 times the baseline stimulation intensity. Experiments were performed for at least 50 minutes after HFS. The amount of potentiation was calculated at 30 minutes after tetanus. Data were filtered at 3 kHz, digitized at 10 kHz, and stored in a computer using pClamp 9.2 software (Axon Instruments, CA, USA). The initial slope of the fEPSPs was measured and plotted against time.

### Western Blotting

Brain protein were collected as previously described [31]. Briefly, proteins were separated on 10% sodium dodecyl sulfate (SDS)-polyacrylamide gels by sodium dodecyl sulfate-polyacrylamide gels electrophoresis (SDS-PAGE); Bio-Rad Laboratories, Foster City, CA, USA. Then, proteins were transferred to polyvinylidene difluoride (PVDF) membrane. PVDF membranes were blocked with 5% non-fat milk in 1×TBST buffer for 1 hour at room temperature. After that, membranes were subsequently exposed to primary antibodies including anti-Bax (Santa Cruz Biotechnology, Santa Cruz, CA, USA), anti-Bcl-2 (Cell Signaling Technology, Danvers, MA, USA), anti-occludin (Santa Cruz Biotechnology), or actin (Cell Signaling Technology) overnight at room temperature, respectively. All membranes were incubated with a secondary goat anti-rabbit antibody, conjugated with horseradish peroxidase (Cell Signaling Technology, Danvers, MA, USA). Finally, the proteins bands were visualized on Amersham hyperfilm enhanced chemiluminescence (ECL) by the Amersham ECL Western blotting detection reagents system (GE healthcare, Buckinghamshire, UK). Total results were shown as average signal intensity.

### Isolation of Brain Mitochondria

Brain mitochondria were isolated using the method described previously [32]. Mitochondrial pellets were collected and dissolved in cold respiration (RES) buffer [33]. The protein concentration was determined according to the Bicinchoninic Acid (BCA) Assay as previously described [34].

### Determination of Brain Mitochondrial Swelling

Brain mitochondrial swelling assay was determined by the changes in the absorbance of the mitochondrial suspensions at 540 nm using a microplate reader [35]. Mitochondria (0.533 mg/ml) were incubated in RES buffer. The decreased in absorbance represented brain mitochondrial swelling.

### Determination of Pharmacological Interventions on Brain Mitochondrial Membrane Potential Changes (ΔΨ_m_)

Brain mitochondrial membrane potential changes were determined using a fluorescent dye, 5, 5′, 6, 6′-tetrachloro-1, 1′, 3, 3′-tetraethylbenzimidazolcarbocyanine iodide (JC-1) [36]. In this protocol, brain mitochondria (0.552 mg/ml) were stained with JC-1 (310 nM) at 37°C for 15 minutes. The fluorescent intensity was determined using a fluorescent microplate reader. The fluorescence of JC-1 monomer form (green) was excited at 488 nm and the emission was detected at 530 nm. JC-1 aggregate (red) fluorescence was excited at 488 nm and emission fluorescence was detected at 590 nm. Mitochondrial depolarization was indicated by a decreased red/green fluorescent intensity ratio (ΔΨm).

### Determination of Brain Mitochondrial ROS Production

Brain mitochondrial ROS production was determined using dichlorohydro-fluorescein diacetate (DCFDA) dye [37]. In this protocol, brain mitochondria (0.552 mg/ml) were incubated with 2-µM DCFDA for 20 minutes. Fluorescence was determined at λex 485 nm and λem 530 nm using a fluorescent microplate reader. The ROS level was represented as arbitrary units of fluorescence intensity of dichlorohydro-fluorescein (DCF).

### Identification of Brain Mitochondria Using Electron Microscopy

A transmission electron microscope was used to study brain mitochondrial morphology. The preparation of brain mitochondria for electron microscope was done according to the method described previously [32], [38]. Briefly, isolated brain mitochondria were fixed overnight in 2.5% glutaraldehyde in 0.1 M cacodylate buffer, pH 7.4 at 4°C. After that, brain mitochondria were postfixed with 1% cacodylate-buffered osmium tetroxide at room temperature for 2 hours. The brain mitochondrial pellets were subsequently dehydrated in a graded series of ethanol and embedded in Epon-Araldite. Ultrathin sections were cut by a diamond knife, then placed on copper grids and stained with uranyl acetate, lead citrate, and observed with a transmission electron microscope.

### Open Field Assessment

Open-field assessment was developed to evaluate the anxiety behavior and general locomotors activity in rodents [39], [40]. Each animal was placed into the box and allowed to spend 5 minutes to explore the box. The numbers of lines which the animals crossed in the peripheral area of the open-field were counted as activity. This parameter was used to exclude the different locomotors activity among rats.

### Morris Water Maze Task (MWM)

MWM was modified from the previous study [41]. The experimental protocol was divided into 2 different phases 1) acquisition test (hidden platform), and 2) probe trial test (removal of the platform from the water pool). In the acquisition test, animals performed 4 different starting points per day for 5 consecutive days. Animals were placed in a randomized starting point with their head turned towards the border of the water pool, the time started recording after placing the animal into the water and stopped recording when the animal found the submerged platform. After that, the animals were placed at the other 3 starting points. All animals performed the probe test on the sixth day. The animals were placed into the water with their head facing towards the water. Time recording starting after placing the animal into the water and the experiment was finished after 90 seconds. Time to reach the platform and time spent in the quadrant which previously contained the platform were recorded.

### Data Analysis

The data for each experiment were presented as mean ± standard error of mean. The data were processed using SPSS (Statistical Package for Social Sciences, Chicago, IL, USA) release 16.0 for Windows. For all comparisons, the significance of the difference between the mean was calculated by Two-way ANOVA, followed by Fisher’s least significant difference *post hoc* analysis. The data from brain mitochondrial function were analyzed using non-parametric Kruskal-Wallis H followed by the Mann-Whitney U test. *P*<0.05 was considered statistically significant.

## Results

### Iron Overload Induced by 4- and 8-week High Iron Diet (HFe) Consumption Increased not only Plasma NTBI Levels and Peripheral Oxidative Stress, but also Brain Iron Accumulation and Brain Oxidative Stress

Four- and eight week high iron (HFe)-fed rats had lower body weights than normal diet (ND)-fed rats (Table 1). We found that both 4- and 8-week HFe-fed rats had iron overload as indicated by increased plasma non-transferrin bound iron (NTBI, Table 1). In addition, increased plasma malondialdehyde (MDA) level was observed in HFe-fed rats (Table 1). HFe-fed rats had high levels of brain iron accumulation and brain oxidative stress as indicated by a significant increase in brain iron and brain MDA level (Table 1). In addition, the iron overload condition, increased oxidative stress, level of brain iron deposition and increased brain oxidative stress occurred in a time-dependent manner of HFe consumption (Table 1).

**Table 1:** Effects of 4-, and 8-week high iron diet consumption on body weight, plasma NTBI, plasma MDA, brain MDA and brain iron level in rats.

Parameters	ND4w	HFe4w	ND8w	HFe8w
Body weight (g)	317.00±3.74	260.83±3.75*	395.00±8.37	305.71±10.99* †
Plasma NTBI (µM)	0	2.51±0.118*	0	3.35±0.141* †
Plasma MDA (µM)	6.42±2.58	23.36±1.72*	10.30±3.46	31.31±3.90* †
Brain MDA (µM/mg protein)	0.83±0.04	1.25±0.04*	1.02±0.10	1.50±0.07* †
Brain iron (µM/mg protein)	7.45±0.13	9.62±0.28*	7.55±0.16	12.91±1.27* †

p<0.05 vs. Normal diet-fed rats,

p<0.05 vs. High iron diet-fed rats for 4 weeks. ND; normal diet-fed rats, HFe; high iron diet-fed rats, 4w; 4-week diet consumption, 8w; 8-week diet consumption, NTBI; non-transferrin bound iron, MDA; malondialdehyde.

### Iron Overload Induced by HFe Led to the Impairment of Learning and Memory and Impaired Brain Synaptic Plasticity

**Figure 2:**
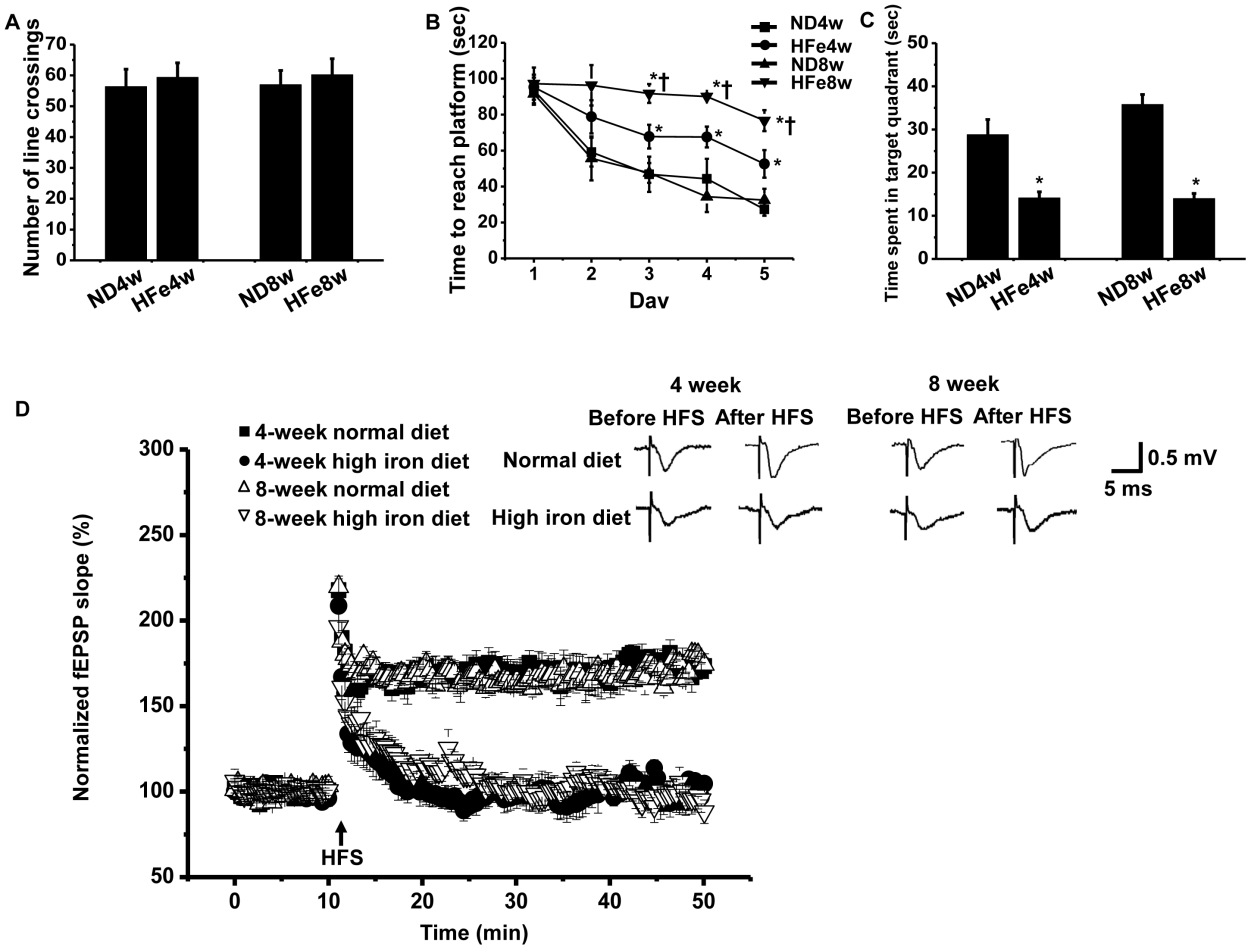
Effects of iron overload on learning and memory behavior. Each panel represented locomotors activity (A) and learning and memory behavior which was indicated by time to reach platform (B) and time spent in target quadrant (C) and brain synaptic plasticity (D). *P<0.05 vs. Normal diet groups; ^†^ *P*<0.05 vs. HFe4w. ND: normal diet fed groups; HFe: high-iron diet fed groups.

High iron diet consumption for 4- and 8-week had no effect on locomotors activity of those rats as indicated by no significant differences in the number of line crossings in rats between both dietary groups in the open field tests (Fig. 2A). However, the iron overload condition caused significant learning and memory deficit as indicated by increased time to reach target platform and decreased time spent in target quadrant of HFe-fed rats compared to those of ND-fed rats during the Morris Water test (*p*<0.05, Fig. 2B and 2C). We also found that the decreased learning process represented by the increase in time to reach a target platform of HFe-fed rats appeared in a time-dependent manner of HFe consumption (Fig. 2B). However, the decreased memory process as indicated by the decrease in time spent in a target quadrant of HFe-fed rats did not occur in a time-dependent manner of HFe consumption (Fig. 2C).

The long term potentiation (LTP) process in CA1 hippocampus was determined in rats of both dietary groups to study brain synaptic plasticity which is an important process for information storage of memory in the brain [42]. After high frequency stimulation (HFS), brain LTP occurred in ND-fed rats, but was absent in HFe-fed rats (3 independent slices/animals, n = 6 animals/group, Fig. 2D). At the 30-minute period after LTP induction, the increments of LTP magnitude compared to baseline in ND-fed rats were 68.72±6.73% for 4-week and 65.79±6.02% for 8-week HFe consumption. However, the LTP magnitudes among these ND groups did not reach statistical difference.

### Brain Mitochondrial Dysfunction in HFe-fed Rats

**Figure 3:**
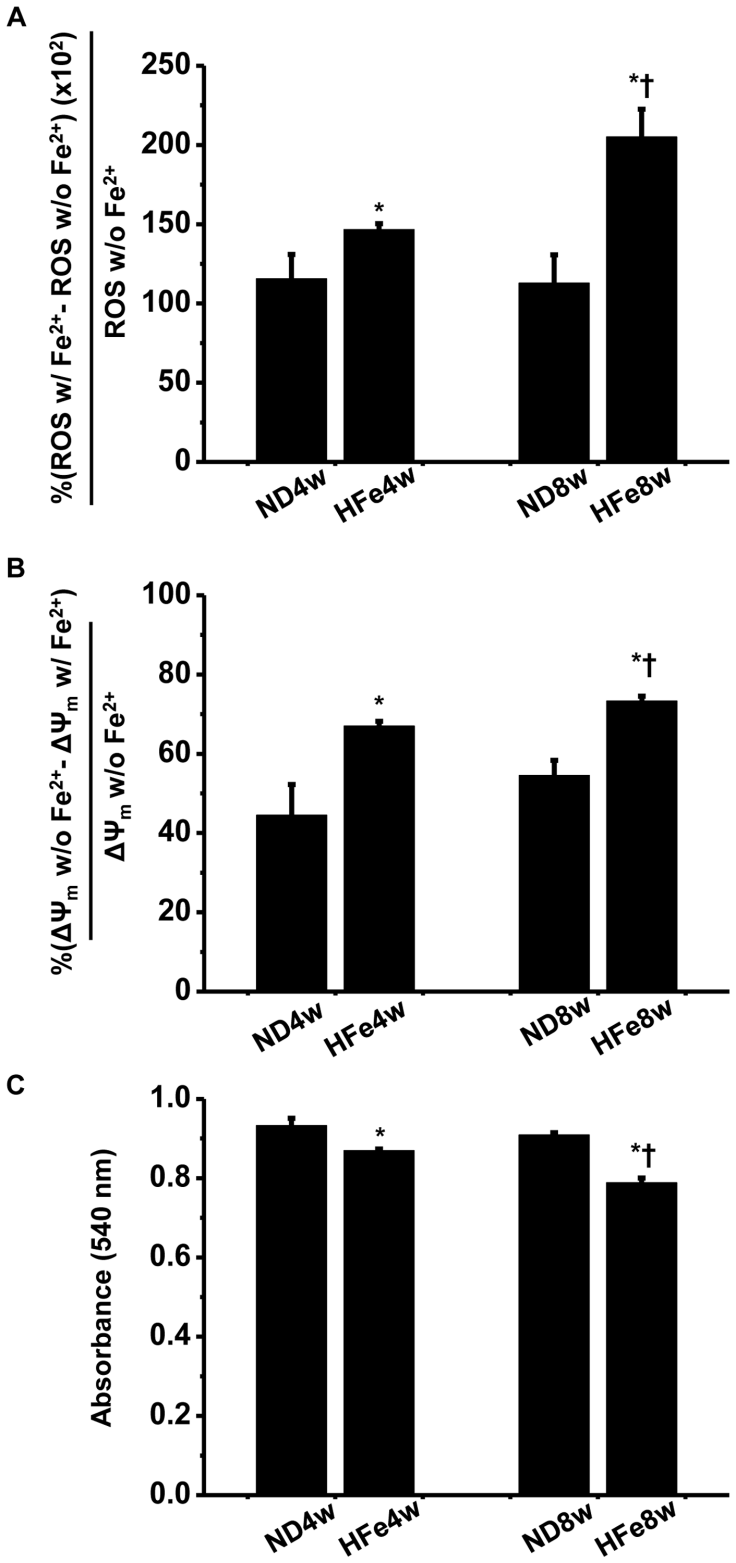
Effects of iron overload on brain mitochondrial function. Each panel represented brain mitochondrial ROS production (A), brain mitochondrial membrane potential changes (B) and brain mitochondrial swelling (C). *P<0.05 vs. Normal diet groups; ^†^ *P*<0.05 vs. HFe4w. ND: normal diet fed groups; HFe: high-iron diet fed groups.

To investigate brain mitochondrial function, brain mitochondrial reactive oxygen species (ROS) production, brain mitochondrial membrane potential change, and brain mitochondrial swelling were determined in both HFe-fed rats and ND-fed rats. We found increased brain mitochondrial ROS production, increased brain mitochondrial depolarization and brain mitochondrial swelling in HFe-fed rats compared with those of ND-fed rats (Fig. 3). This mitochondrial dysfunction also occurred in a time-dependent manner of HFe consumption (Fig. 3).

### HFe Consumption Leads to the Blood-brain Barrier Breakdown and Brain Apoptosis

**Figure 4:**
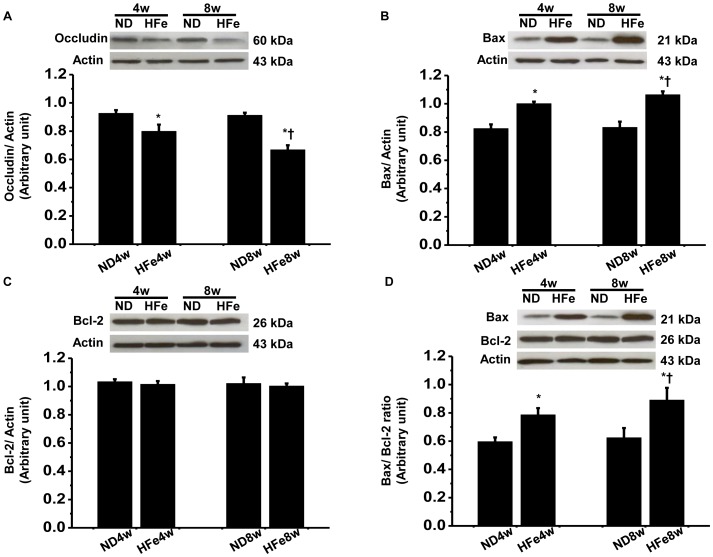
Effects of iron overload on BBB breakdown and apoptosis. Each panel represented the expression of tight junctions protein; occludin (A), apoptotic-protein; Bax (B) and anti-apoptotic protein; Bcl-2 (C) and Bax/Bcl-2 ratio (D). *P<0.05 vs. Normal diet groups; ^†^ *P*<0.05 vs. HFe4w. ND: normal diet fed groups; HFe: high-iron diet fed groups.

The expression of occludin, which is the tight junction marker in cerebral endothelial cells was determined to investigate the blood-brain barrier (BBB) breakdown [43], [44]. HFe-fed rats had decreased expression of occludin, compared to ND rats (Fig. 4A). Moreover, the expression of occludin in 8-week HFe-fed rats was significantly lower than that in 4-week HFe-fed rats, suggesting that the BBB breakdown induced by iron overload occurred in a time-dependent manner of HFe consumption.

Although HFe consumption had no effect on the expression of anti-apoptotic marker Bcl-2, the expression of apoptotic marker Bax was markedly increased (Figure 4B-4C). The ratio of Bax/Bcl-2 was also significantly increased, compared to those of ND-fed rats (Fig. 4D). Both BBB breakdown and increased apoptotic marker occurred in a time-dependent manner of HFe consumption.

Although either iron chelator deferiprone (L1) or anti-oxidant n-acetyl cysteine (NAC) attenuated the iron overload condition as well as brain dysfunctions, their combined therapy restored those defects induced by iron overload.

#### Effects of L1 and NAC on peripheral and brain parameters in iron overload condition

Treatment with either L1 or NAC alone significantly attenuated the adverse effects of iron overload condition in HFe-fed rats as indicated by increased body weight, and decreased plasma NTBI and plasma MDA levels. No differences of these parameters were found between both therapies. However, despite the reduction of plasma NTBI and MDA by either treatment, they did not reach the normal levels as shown in ND-fed rats (Table 2). Similarly, brain MDA level and brain iron level of HFeL1 rats and HFeNAC rats were also significantly decreased, compared to those of HFeV rats, but did not reach the normal levels as shown in the ND group (Table 2). However, the combination of L1 and NAC treatment (HFeL1+NAC) completely restored plasma NTBI, plasma MDA level, brain MDA level and brain iron level in HFe-fed rats to the same levels as shown in ND group (*p*<0.05, Table 2).

**Table 2:** Effects of the pharmacological interventions on body weight, plasma NTBI, plasma MDA, brain MDA and brain iron level in high iron diet-fed rats.

Parameters	NDV	NDL1	NDNAC	HFeV	HFeL1	HFeNAC	HFeL1+NAC
Body weight (g)	433.33±18.56	401.67±37.56	411.67±13.02	325.00±9.92*	358.33±19.26* †	362.5±11.60* †	366.67±11.12* † ‡
Plasma NTBI (µM)	0	0	0	3.99±0.51*	0.44±0.19* †	0.54±0.37* †	0
Plasma MDA (µM)	26.01±2.62	26.93±4.47	27.48±3.79	60.76±1.81*	54.75±2.30* †	55.41±2.12* †	31.11±1.6† ‡
Brain MDA (µM/mg protein)	0.97±0.12	0.85±0.16	0.73±0.12	1.71±0.05*	1.30±0.15* †	1.25±0.05* †	0.81±0.08† ‡
Brain iron (µM/mg protein)	8.30±0.52	7.78±0.367	7.99±0.49	23.59±3.33*	13.29±1.23* †	13.70±1.67* †	8.50±0.37† ‡

p<0.05 vs. Normal diet-fed rats,

p<0.05 vs. High iron diet-fed rats treated with vehicle,

p<0.05 vs. High iron diet-fed rats treated with either L1 or NAC. ND; normal diet-fed rats, HFe; high iron diet-fed rats, V; vehicle, L1; deferiprone, NAC; n-acetyl cysteine, NTBI; non-transferrin bound iron, MDA; malondialdehyde.

#### L1 and NAC attenuated learning and memory deficit, and the impairment of brain synaptic plasticity following iron overload condition

**Figure 5:**
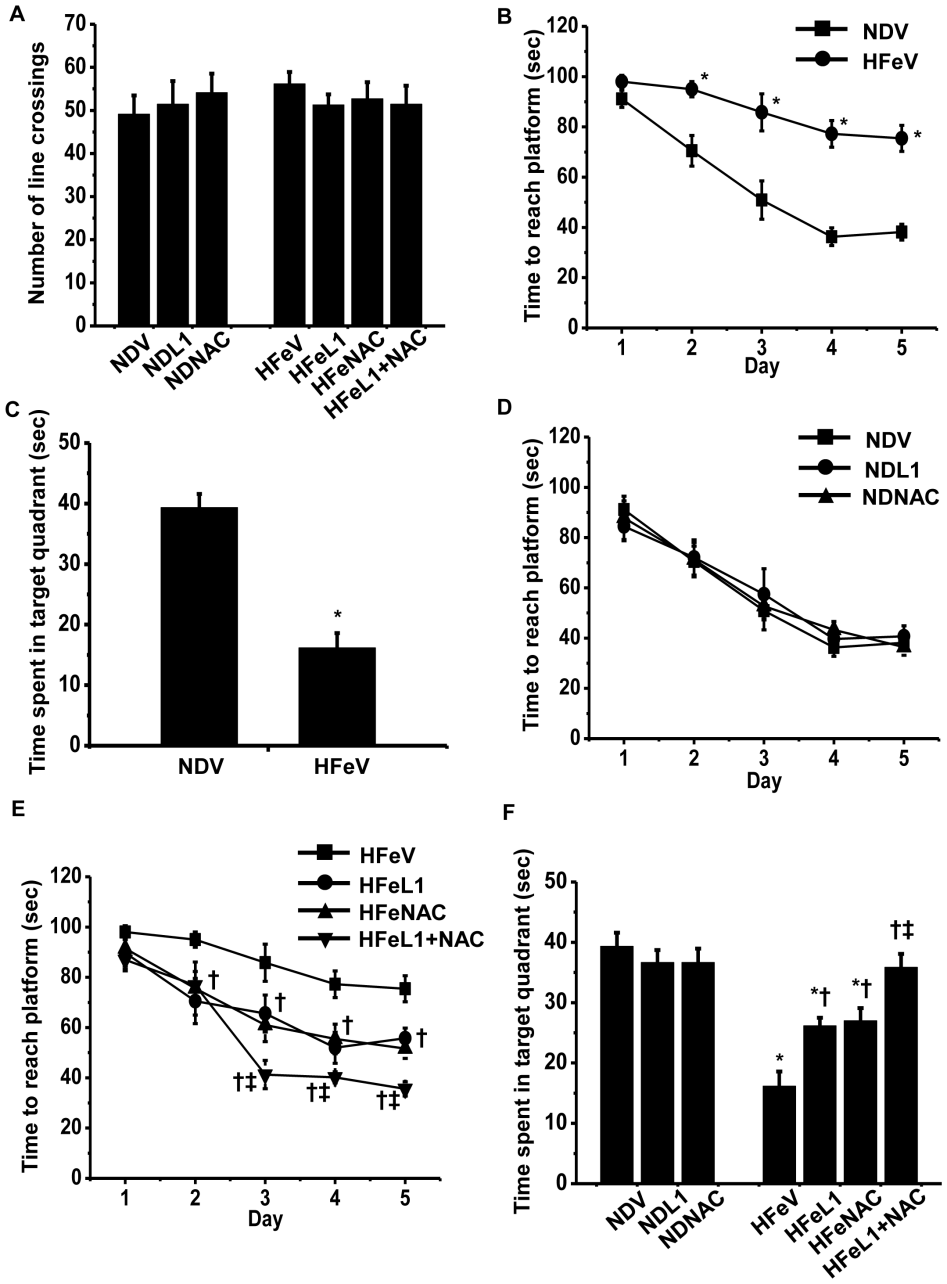
Effects of the pharmacological interventions by deferiprone and n-acetyl cysteine on learning and memory behavior. Each panel represented locomotors activity (A) and learning and memory behavior indicated by time to reach platform (B, D and F) and time spent in target quadrant (C and E). *P<0.05 vs. Normal diet treated with vehicle (NDV); ^†^ *P*<0.05 vs. HFe treated with vehicle (HFeV); ^‡^ *P*<0.05 vs. HFe treated with either L1 or NAC (HFeL1 or HFeNAC). ND: normal diet fed groups; HFe: high-iron diet fed groups; L1: deferiprone; NAC: n-acetyl cysteine.

Although HFe consumption for 12 weeks led to altered peripheral parameters including decreased body weight, increased plasma NTBI and plasma MDA as well as increased brain MDA level and accumulated brain iron level (Table 2), it did not affect the locomotors activity (Fig. 5A).

In the vehicle-treated rats, 12-week HFe consumption required significantly increased time to reach a platform and decreased time spent in a target quadrant, compared to those of ND-fed rats, suggesting the learning and memory deficit following HFe consumption (Fig. 5B and 5C). Neither L1nor NAC treatment alone changed the learning and memory in ND-fed rats (Fig. 5D and 5F). In HFe-fed rats, either L1 or NAC therapy alone significantly improved learning and memory as shown by decreased time to reach a platform, and increased time spent in a target quadrant (Fig. 5E and 5F). In addition, the combined therapy of L1 and NAC completely restored brain functions that were impaired by HFe by improving learning and memory process (Fig. 5E and 5F).

**Figure 6:**
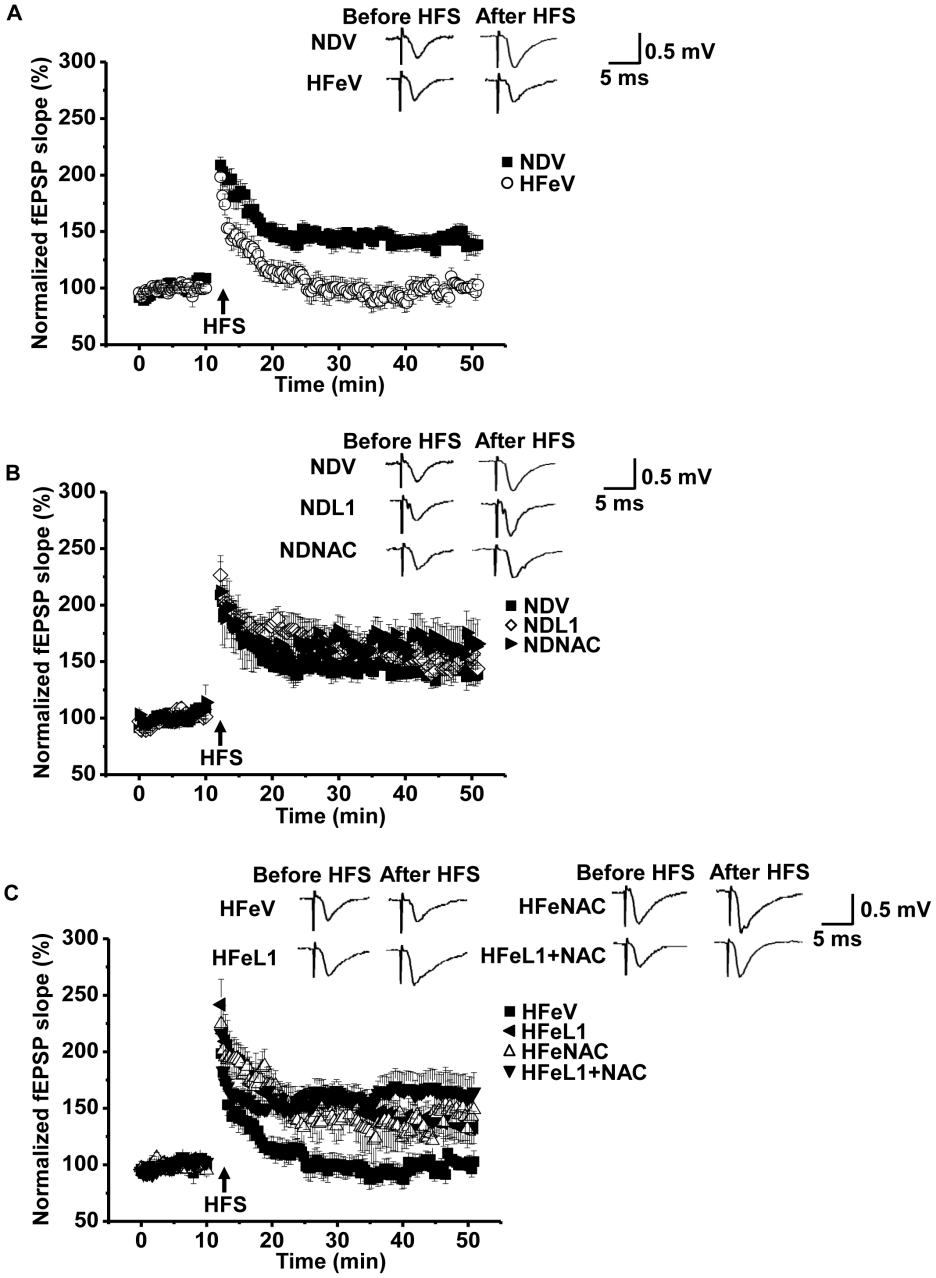
Effects of the pharmacological interventions by deferiprone and n-acetyl cysteine on brain synaptic plasticity. Each panel represented brain synaptic plasticity in NDV vs. HFeV (A), ND groups (B) and HFe groups (C). ND: normal diet fed group; HFe: high iron diet fed group; L1: deferiprone; NAC: n-acetyl cysteine.

Rats in the 12-week HFe consumption also had impaired brain synaptic plasticity as shown by the absent of an electrical-induced LTP after electrical stimulation (3 independent slices/animals, n = 6 animals/group, Fig. 6A). Moreover, treatment with either L1 or NAC alone did not alter the electrical-induced LTP in ND-fed rats (Fig. 6B). At 30 minutes following the LTP induction, the increment of LTP magnitude compared to baseline in ND-fed rats were 61.68±2.85%, 63.57±13.52% and 66.05±13.52% for NDV, NDL1 and NDNAC, respectively (3 independent slices/animals, n = 6 animals/group). Either L1 or NAC alone significantly attenuated the impaired LTP in HFe-fed rats (Fig. 6C). At 30 minutes following the LTP induction, the increment of LTP magnitude compared to baseline were 39.12±8.81% for HFeL1 and 34.10±3.45% for HFeNAC. The increased LTP magnitude between HFeL1 and HFeNAC groups was significantly increased compared to that of HFe-fed rats treated with vehicle.

In addition, combined treatment of L1 and NAC in HFe-fed rats restored the impairment of brain synaptic plasticity (3 independent slices/animals, n = 6 animals/group, Fig. 6C). At 30 minutes after the LTP induction, the increment of LTP magnitude compared to the baseline in the HFeL1+NAC group was 67.36±10.51%, which was significantly increased compared to that of HFeV rats, HFeL1 rats and HFeNAC rats (Fig. 6C).

#### L1 or NAC improved brain mitochondrial dysfunction caused by iron overload condition

**Figure 7:**
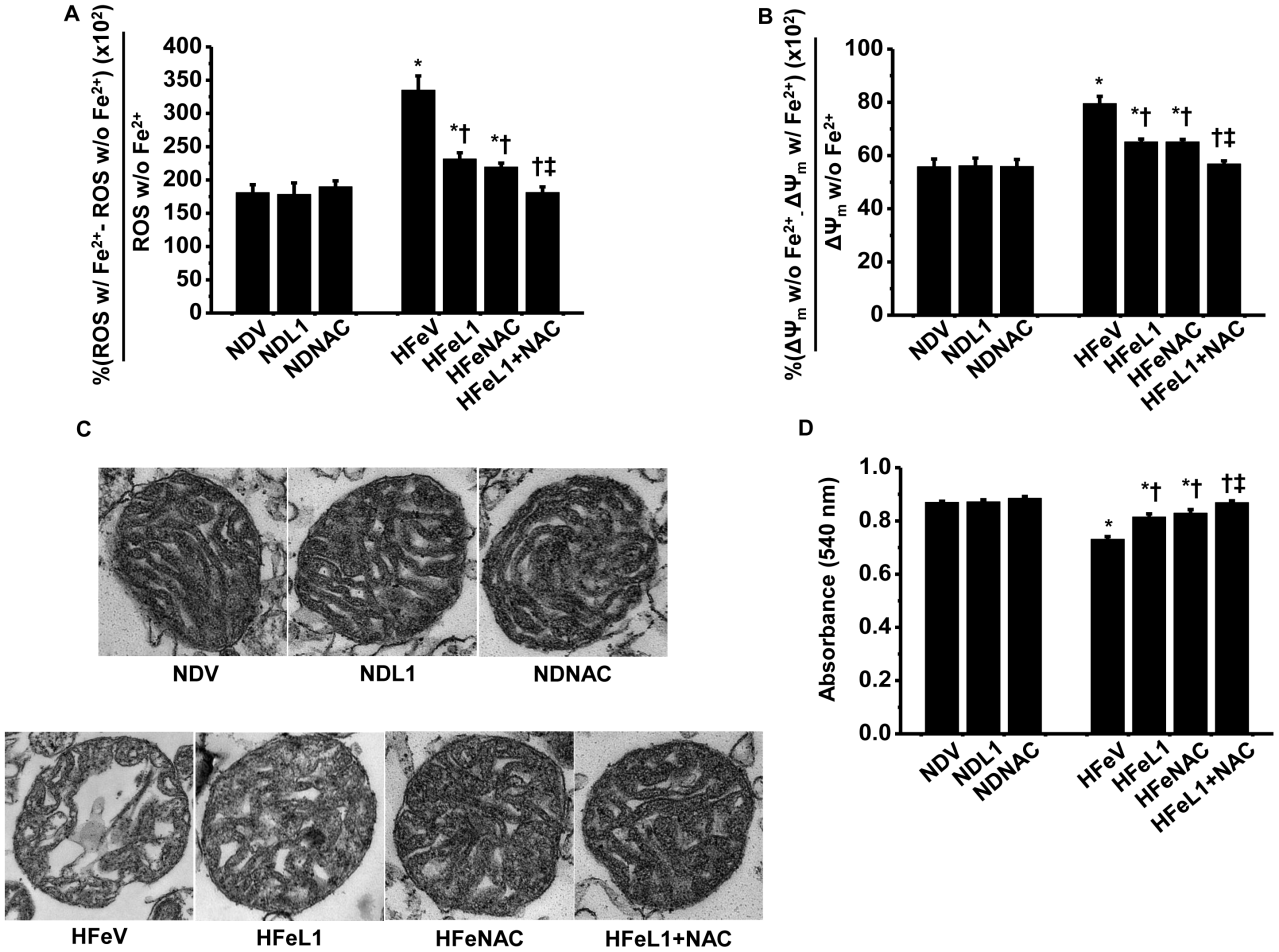
Effects of the pharmacological interventions by deferiprone and n-acetyl cysteine on brain mitochondrial function. Each panel represented brain mitochondrial ROS production (A), brain mitochondrial membrane potential changes (B), brain mitochondrial morphological changes (C) and brain mitochondrial swelling (D). *P<0.05 vs. Normal diet treated with vehicle (NDV); ^†^ *P*<0.05 vs. HFe treated with vehicle (HFeV); ^‡^ *P*<0.05 vs. HFe treated with either L1 or NAC (HFeL1 or HFeNAC). ND: normal diet fed groups; HFe: high-iron diet fed groups; L1: deferiprone; NAC: n-acetyl cysteine.

Twelve week HFe consumption led to brain mitochondrial dysfunction as indicated by increased brain mitochondrial ROS production, brain mitochondrial depolarization, and brain mitochondrial swelling (Fig. 7). HFe-fed rats treated with either L1 or NAC significantly preserved brain mitochondrial function in HFe-fed rats, compared to that of HFe-fed rats treated with vehicle (Fig. 7). Moreover, electron microscopic images revealed that brain mitochondria in HFe-fed rats had altered morphology including unfolded cristae, compared to the normal brain mitochondria, indicating brain mitochondrial swelling (Fig. 7C). Treatment with either L1 or NAC alone in HFe-fed rats attenuated brain mitochondrial dysfunction as shown by decreased mitochondrial ROS level (Fig. 7A), decreased membrane potential change (Fig. 7B), and reduced mitochondrial swelling caused by iron overload (Fig. 7C and 7D). However, the combination of L1 and NAC therapy in HFe-fed rats restored brain mitochondrial function and morphology, compared to that of HFe-fed rats treated with vehicle or treated with either L1 or NAC alone (Fig. 7).

#### L1 or NAC improved the blood-brain barrier breakdown and brain apoptosis caused by iron overload

**Figure 8:**
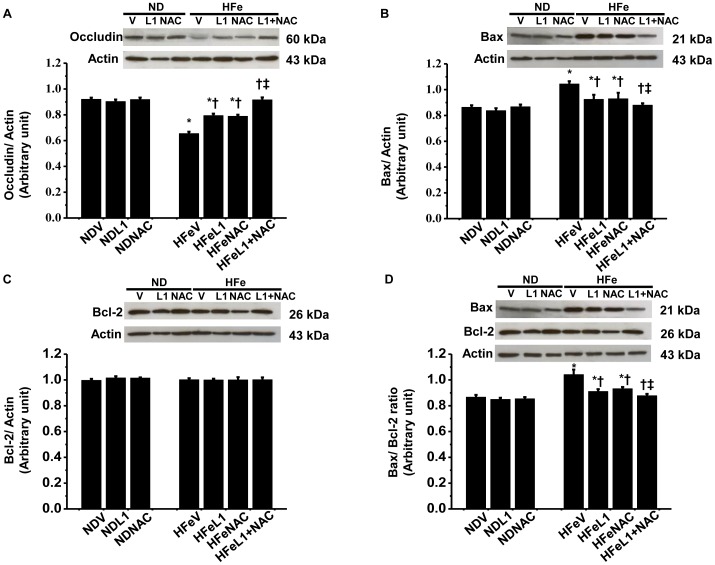
Effects of the pharmacological interventions by deferiprone and n-acetyl cysteine on BBB breakdown and apoptosis. Each panel represented the expression of tight junction protein; occludin (A), apoptotic-protein; Bax (B) and anti-apoptotic protein; Bcl-2 (C) and Bax/Bcl-2 ratio (D). *P<0.05 vs. Normal diet treated with vehicle (NDV); ^†^ *P*<0.05 vs. HFe treated with vehicle (HFeV); ^‡^ *P*<0.05 vs. HFe treated with either L1 or NAC (HFeL1 or HFeNAC). ND: normal diet fed groups; HFe: high-iron diet fed groups; L1: deferiprone; NAC: n-acetyl cysteine.

Both L1 and NAC administration in HFe-fed rats significantly increased the expression of occludin, compared to that of HFe-fed rats treated with vehicle (Fig. 8A). In addition, either L1 or NAC therapy alone also significantly decreased the expression of Bax, although the expression of Bcl-2 in HFe-fed rats was not altered in any groups compared to the control (Fig. 8B and 8C). The Bax/Bcl-2 ratio was also decreased in the L1 and NAC-treated groups (Fig. 8D).

Interestingly, the combination of L1 and NAC therapy restored the occludin expression in HFe-fed rats, suggesting the prevention of BBB breakdown (Fig. 8A). Furthermore, it also attenuated the expression of apoptosis markers, indicating the protective effects of these combined drugs against brain apoptosis caused by HFe consumption (Fig. 8B and 8D).

## Discussion

The major findings of the present study are as follows: 1) Long-term HFe consumption caused iron overload by increasing plasma NTBI and brain iron level, and led to peripheral and brain oxidative stress, 2) HFe induced iron overload was time-dependent of HFe consumption, 3) Iron overload led to learning and memory deficit, abolished brain synaptic plasticity, brain apoptosis, and caused brain mitochondrial dysfunction as indicated by increased brain mitochondrial ROS production, brain mitochondrial depolarization and brain mitochondrial swelling, 4) Brain iron-overload following HFe consumption occurred via the blood-brain barrier breakdown, 5) The pharmacological intervention using either deferiprone or n-acetyl cysteine alone attenuated these deleterious effects of iron overload and 6) The present study is the first study that demonstrated that the combination of deferiprone and n-acetyl cysteine exerted the robust effects by restoring brain function following iron overload.

In the present study, chronic HFe consumption induced iron-overload condition as indicated by an increased plasma non-transferrin bound iron (NTBI). The plasma NTBI is known to be catalyzed, resulting in the production of highly toxic free hydroxyl radicals via Fenton reactions [45], [46]. These excessive free radicals have been shown to cause cellular lipid peroxidation and increased cytotoxic aldehyde products including 4-hydroxylnonenal (HNE) and malondialdehyde (MDA) [10], [47]. Consistent with these reports, we found that in rats fed with HFe increased plasma NTBI were observed with increased plasma MDA. In the present study, increased brain iron accumulation in HFe rats was also observed. It is known that plasma iron could not normally pass the intact blood-brain barrier (BBB) since the BBB is a diffusion barrier playing a role in brain defensive mechanism [48]. Under several pathological conditions, the decrease of tight junction protein occludin could be observed, indicating the BBB disruption [43], [44]. Won and colleagues demonstrated in a brain injury model that decreased expression of tight junction proteins could be observed, causing an increased BBB permeability, and eventually leading to increased brain iron accumulation [49]. In the present study, increased occludin expression was observed, indicating the BBB disruption and could be responsible for increased brain iron accumulation in HFe rats. In addition, increased brain iron level could also cause further lipid peroxidation in the brain as shown by increased brain MDA level in our study.

Mitochondria are vital organelles since they are the power houses of cells [50]. Although iron is required as a cofactor in the oxidative phosphorylation process in the mitochondria for ATP production, excessive iron could cause severe oxidative stress including ROS and MDA levels, which is harmful to the cells [51]–[55] and mitochondria [32], [51], [53], [56]–[58]. As demonstrated in the present study, HFe rats had high levels of brain iron, leading to brain oxidative stress and brain mitochondrial dysfunction as indicated by increased brain mitochondrial ROS production, brain mitochondrial depolarization and brain mitochondrial swelling. It has been shown that mitochondrial dysfunction is a primary cause of the cellular apoptosis [59]–[61]. Consistent with these reports, we found that iron overload in the brain increased brain apoptosis as shown by increased expression of Bax and Bax/Bcl-2 ratio without changing the Bcl-2 expression. These findings suggested that iron overload causes brain mitochondrial dysfunction, leading to brain apoptosis.

It has been known that iron overload leading to iron toxicity could be observed in several organs particularly in the brain [10], [62]–[66]. Several studies showed that iron toxicity in the brain caused memory deficit [65]–[67]. In the present study, chronic HFe consumption induced iron overload also led to learning and memory deficit as shown in the Morris Water Maze test. Consistent with the learning and memory deficit, we also found that the brain synaptic plasticity, the important process of the learning and memory [42], [68], was impaired following iron overload condition. These findings indicated that iron overload condition could cause cognitive impairment, and that brain mitochondrial dysfunction would be the primary target responsible for this adverse effect.

Although HFe-fed rats showed the significant reduction of their body weight, compared to ND-fed rats (Table 1), this reduction of body weight in HFe-fed rats did not cause the alteration in muscle activity during Morris water maze test. This is due to the fact that the locomotors activity as well as the time to reach the target platform between ND-fed rats and HFe-fed rats on the 1^st^ and 2^nd^ day of the acquisition test (after iron overload) were not significantly different (Fig. 2A), indicating that the muscle activity for swimming of both groups was similar. Thus, increased time to reach the target platform on the 3^rd^-5^th^ day of the acquisition test, and decreased time spent in the target quadrant on the 6^th^ day of probe test in HFe-fed rats compared to ND-fed rats during the Morris Water test (as shown in Fig. 2B and 2C) should directly indicate the impairment of learning and memory.

Surprisingly, we noticed that the defective “learning” process, as measured by the acquisition test, brain mitochondrial dysfunction and increased brain apoptotic markers were exhibited in a time-dependent manner of HFe consumption. However, the defective “memory” process, as measured by the probe test and the impairment of brain synaptic plasticity were not exhibited in a time-dependent manner of HFe consumption. These findings suggested that 1) The learning process in the brain required certain amounts of healthy brain mitochondria and healthy neurons to be able to function, and 2) The memory process was associated with the brain synaptic plasticity. Since the brain synaptic plasticity is an essential process of the memory formation [69], our findings indicated that iron overload not only impaired the learning process, but also destroyed the memory process.

Although pharmacological intervention of iron overload has been widely used, the effects of pharmacological interventions on brain dysfunction following iron overload have been rarely investigated. Iron chelators such as deferiprone (L1) and deferoxamine were widely used to attenuate the harmful effects from iron overload condition in thalassemic patients [70], [71]. However, L1 has an advantage over deferoxamine since it could be orally administered whereas deferoxamine administration needed to be injected. Several studies demonstrated the potential effect of iron chelation therapy in brain injury [25], [26]. Those findings implied that iron chelation therapy might be useful in attenuating brain dysfunction induced by iron overload. Consistent with this concept, our study demonstrated that L1 could attenuate brain dysfunction from iron overload.

Since oxidative stress from iron overload was also responsible for brain dysfunction, another potential strategy to protect the brain from iron-overload induced oxidative stress would be an anti-oxidative therapy. N-acetyl cysteine (NAC) has been commonly used as an antioxidant [72]. Several studies suggested the beneficial effect of NAC in oxidative stress by decreasing ROS production [21], [22]. In the present study, NAC administration alone in HFe-fed rats could attenuate brain dysfunction in a similar fashion observed with L1 therapy. These findings suggest that both iron chelator and antioxidant agent alone provided the beneficial effects in iron overload induced brain dysfunction. Nevertheless, neither L1 nor NAC treatment alone could completely restore the brain function following iron overload.

**Figure 9:**
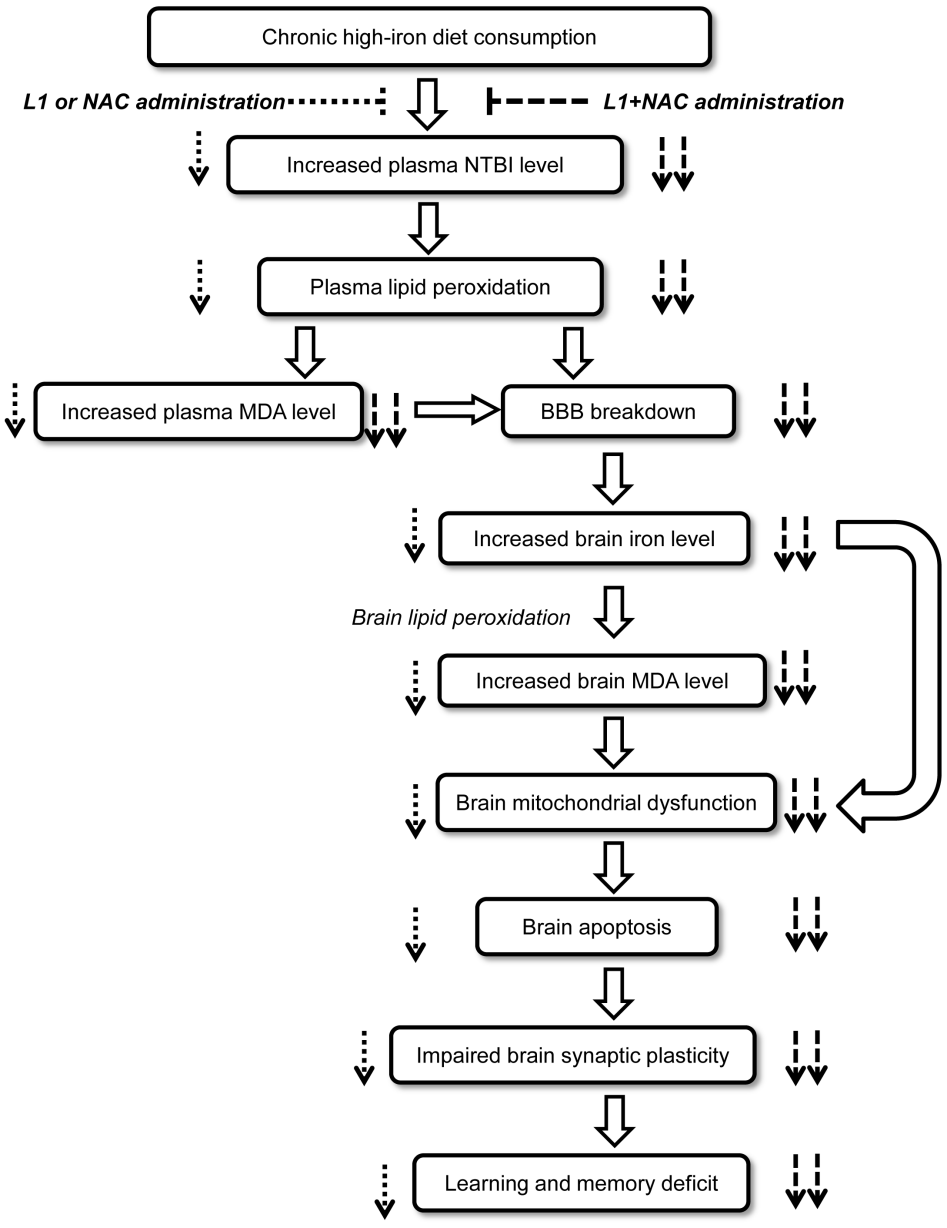
Diagram illustrated the proposed mechanisms. The diagram showed the proposed mechanisms of brain dysfunctions as well as the pharmacological therapy on brain dysfunctions following iron overload. Dotted arrows indicate the effects of either L1 or NAC treatment, dashed arrows indicate the combined L1 and NAC treatment. L1: deferiprone; NAC: n-acetyl cysteine.

Despite the concept that both antioxidant and iron chelation would be essential in treating iron overload, the effects of combination of both agents in treating brain dysfunction due to HFe-induced iron overload have been rarely investigated. We further explored whether the combination of L1 and NAC treatment on brain function following iron overload provided more beneficial effects than the single agent therapy. Interestingly, we demonstrated for the first time that combined therapy of L1 and NAC completely restored brain function impaired by long-term HFe consumption. The possible mechanisms of the combination therapy to recover brain dysfunction induced by iron overload would be due to several beneficial effects provided by each agent. First, combined therapy could decrease iron accumulation via L1, and decrease iron-induced oxidative-stress by NAC as well as L1, which was previously shown to act as an anti-oxidant agent [73]. Second, both L1 and NAC had been shown to have direct effects on the brain since both drugs could pass the blood brain barrier [74], [75]. As a result, the combined therapy could provide dual beneficial effects in both plasma and directly in the brain. As demonstrated for the first time by our study, the combined therapy provided a greater therapeutic benefit by restoring brain function impaired by iron overload. Future clinical studies are needed to confirm the beneficial effects of the combined L1 and NAC observed in the present report. Summarized proposed mechanisms including brain dysfunctions following iron overload, and the effects of pharmacological interventions on attenuating brain dysfunction are illustrated in figure 9.

### Conclusions

The present study demonstrated the deleterious effects of iron overload in the brain as presented by learning and memory deficit, inhibition of brain synaptic plasticity, brain mitochondrial dysfunction and brain apoptosis. Iron overload condition led to blood-brain barrier breakdown causing an increased brain iron level and consequently causing brain dysfunction. Pharmacological interventions by either iron chelator; L1 or an antioxidant; NAC attenuated iron overload-induced brain dysfunctions. However, combined L1 and NAC not only attenuated but also restored brain dysfunctions impaired by long-term HFe consumption. The present study is the first study to show that the combined therapy with L1 and NAC provided the greater beneficial effects in improving brain dysfunction caused by iron overload.

### Study Limitations

Although our study suggested that brain iron overload led to brain dysfunctions and the pharmacological interventions using L1 and NAC showed the beneficial effects on brain functions, the effects of brain iron overload on the metabolism of other metal ions such as Zn^2+^ and Cu^2+^, and the effects of combined therapy on brain function under the physiological condition was not investigated in the present study. Moreover, high iron level is known to be associated with the infection and inflammation. Previous studies reported that under iron overload condition, thalassemic patients were susceptible to the microbial infection [76]–[78]. In addition, previous study showed that iron overload could also lead to systemic inflammation [79]–[83]. Recent studies showed that systemic inflammation under several pathological conditions could lead to BBB dysfunction as well as microbial infection [82], [84]–[89]. All of these findings suggested that iron overload was associated with infection and inflammation, which could have influenced BBB integrity and led to BBB dysfunction. Future studies are needed to clarify these mechanisms related to BBB dysfunction as well as the BBB permeability under this pathological condition.
